# Structural bioinformatics predicts that the Retinitis Pigmentosa-28 protein of unknown function FAM161A is a homologue of the microtubule nucleation factor Tpx2

**DOI:** 10.12688/f1000research.25870.1

**Published:** 2020-08-27

**Authors:** Timothy P. Levine

**Affiliations:** 1 UCL Institute of Ophthalmology, University College London, London, EC1V 9EL, UK

**Keywords:** Structural Bioinformatics, Microtubule Associated Protein, Centrosome, Primary Cilium, Retinitis Pigmentosa, Nucleation Factor, Tpx2, FAM161A

## Abstract

**Background:** FAM161A is a microtubule-associated protein conserved widely across eukaryotes, which is mutated in the inherited blinding disease Retinitis Pigmentosa-28. FAM161A is also a centrosomal protein, being a core component of a complex that forms an internal skeleton of centrioles. Despite these observations about the importance of FAM161A, current techniques used to examine its sequence reveal no homologies to other proteins.

**Methods:** Sequence profiles derived from multiple sequence alignments of FAM161A homologues were constructed by PSI-BLAST and HHblits, and then used by the profile-profile search tool HHsearch, implemented online as HHpred, to identify homologues. These in turn were used to create profiles for reverse searches and pair-wise searches. Multiple sequence alignments were also used to identify amino acid usage in functional elements.

**Results:** FAM161A has a single homologue: the targeting protein for *Xenopus* kinesin-like protein-2 (Tpx2), which is a strong hit across more than 200 residues. Tpx2 is also a microtubule-associated protein, and it has been shown previously by a cryo-EM molecular structure to nucleate microtubules through two small elements: an extended loop and a short helix. The homology between FAM161A and Tpx2 includes these elements, as FAM161A has three copies of the loop, and one helix that has many, but not all, properties of the one in Tpx2.

**Conclusions:** FAM161A and ­its homologues are predicted to be a previously unknown variant of Tpx2, and hence bind microtubules in the same way. This prediction allows precise, testable molecular models to be made of FAM161A-microtubule complexes.

## Introduction

Inherited eye disease affects approximately 1 in 1500 people in Western societies. The largest grouping within these disorders is Retinitis Pigmentosa (RP) ( Daiger *et al.*, 2013). RP is itself a diverse array of conditions that share a final common pathway of loss of photoreceptor function, usually with initial loss of rod photoreceptors diminishing peripheral and night vision, followed by critical central vision defects in both rods and cones.

Mutations in at least 56 genes cause inherited RP syndromes ( Daiger *et al.*, 2013). Some of the proteins coded by genes mutated in RP, such as rhodopsin, are obviously linked to photoreceptor function ( Athanasiou *et al.*, 2018). Other RP proteins have general functions in many cells, for example in the mRNA spliceosome, and studies of these forms of RP have revealed how specific house-keeping pathways are critical for photoreceptor function ( Ruzickova & Stanek, 2017). A small, third group of RP proteins have remained mysterious because no molecular function can be assigned. The lack of information stems from these proteins having no domains with recognisable functional activities. The failure of routine methods to identify domains exists not only for RP proteins, but also across the human proteome. Even the most highly annotated proteome, the budding yeast *Saccharomyces cerevisiae*, has a substantial minority of proteins (20%) without useful functional information ( Wood *et al.*, 2019).

Among 56 proteins linked to RP, functional information is missing in several, including FAM161A, truncations of which cause recessive RP28 ( Bandah-Rozenfeld *et al.*, 2010; Langmann *et al.*, 2010). FAM161A binds to microtubules ( Zach *et al.*, 2012), and localises to the primary cilium ( Di Gioia *et al.*, 2012; Estrada-Cuzcano *et al.*, 2012; Nishimura *et al.*, 2010; Zach *et al.*, 2012), which is vital in photoreceptor function through its specialisation as the outer segment. FAM161A is also a centrosomal protein ( Di Gioia *et al.*, 2015), and is a key component of a complex with three other proteins that together form an internal framework for centriolar microtubules ( Le Guennec *et al.*, 2020).

FAM161A has retained its systematic name in part because no function has been detectable from its sequence. The region of greatest conservation in FAM161A has been annotated as UPF0564, one of >10,000 **d**omains of **u**nknown **f**unction (DUFs) and **u**ncharacterized **p**rotein **f**amilies (UPFs) ( Bateman *et al.*, 2010). It has also been named PF10595 by the protein families (PFAM) project ( Finn *et al.*, 2016). While analysis of UPF0564/PF10595 shows that FAM161 proteins are distributed across eukaryote evolution, the lack of any homologues of known function, means that the function of the entire protein family remains unknown.

The question of proteins of unknown function would be addressed in large measure by determining their structure, which narrows the range of possible functions ( Lobb & Doxey, 2016; Zhang *et al.*, 2014). In the absence of solved structure, an interim measure is to predict domain structures using structural bioinformatics, one major branch of which is remote homology sensing of distant homologies between DUFs and domains of known function ( Bateman *et al.*, 2010). Following the widespread application of sequence-sequence comparison tools such as BLAST ( Altschul *et al.*, 1990), search tools were made more sensitive by adding iterations where the output of one search is submitted as the query for the next. This creates a protein alignment that represents multiple homologues in a profile that carries information about sites of conservation and variation, including where deletions and insertions are tolerated. Iterative tools such as PSI-BLAST perform profile-sequence searches, which find homologues with greater sensitivity ( Altschul *et al.*, 1997). A further step-change increase is to use profiles on both sides ( *i.e.* both as query and as target), which is described as a profile-profile search, which requires the preparation in advance of large libraries of profiles (for example: all human proteins, or all PFAM entries) ( Yona & Levitt, 2002).

This article describes sequence-sequence, profile-sequence, and profile-profile searches that strongly predict FAM161A is a homologue of Targeting Protein for Xklp2 (TPX2). The implication is that FAM161 homologues across evolution act as microtubule nucleation factors.

## Methods

Searches were carried out at the Tuebingen Toolkit, unless otherwise stated ( Zimmermann *et al.*, 2018). FAM161A isoform 2 (660 aa, accession number Q3B820) was used in searches, and numbering is for this isoform. Likewise, the Tpx2 sequence used is isoform X1 (747 aa, Q9ULW0).

PSI-BLAST was carried out querying the nr30 or nr50 databases (sequence redundancy reduced so that no sequence shares more than 30% and 50% identity with any other respectively), with inclusion threshold e-value =0.001 for forwarding to subsequent rounds. PSI-BLAST with low complexity filter was carried out at NCBI using the Refseq database (nr100, but 40% smaller than the database called “nr”). HHblits was used iteratively in the same way, except all queries used the nr30 database. Structural predictions were made by PSI-PRED 3.0 within HHpred ( Alva *et al.*, 2016; Zimmermann *et al.*, 2018). HHpred was used with default settings, in particular with the Maximum Accuracy realignment off, unless otherwise stated, in which case the default threshold of 0.3 was chosen. Note that all search results exclude hits with pSS <10%, so these weak hits cannot be reported. Simple alignment of two sequences was carried out in NCBI BLASTP with standard settings. To align two proteins with maximal sensitivity, pairwise HHpred profile-profile searches were carried out by replacing the target library with a single converged target profile. To visualise alignments of small regions, multiple sequence alignments were pruned in Jalview by hand to reduce redundancy and fragmentary sequences. Logos were drawn with Weblogo ( Crooks *et al.*, 2004). Sequences not already aligned by PSI-BLAST, HHblits or HHpred were aligned with MAFFT. 

## Results

FAM161A is homologous to the microtubule nucleation factor Tpx2

### Building profiles

**Figure 1.**
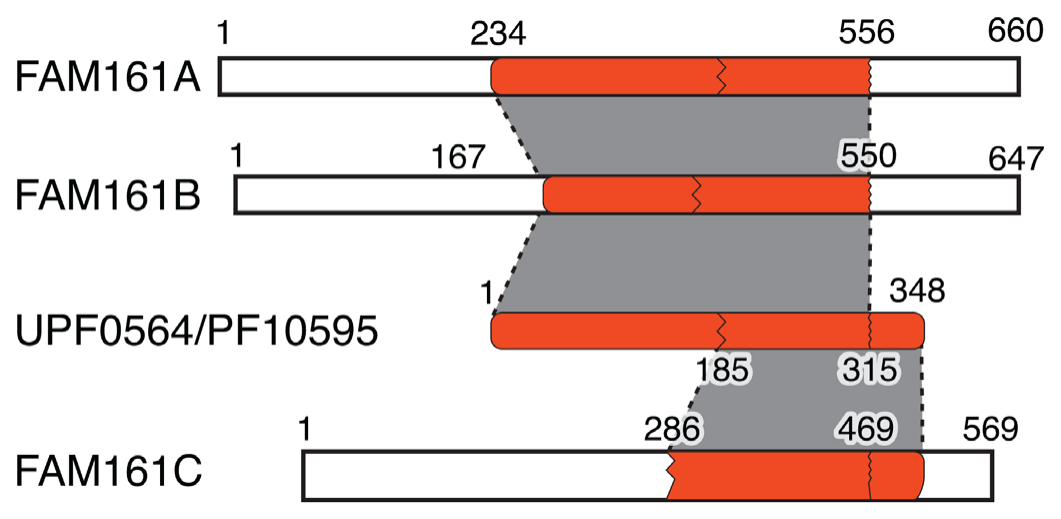
Alignment of human FAM161A/B/C with UPR0564/PF10595. Alignments include small gaps (up to 60 residues) that are not indicated. No protein contains 100% of UPR0564/PF10595. Regions of proteins that align with UPR0564/PF10595 are indicated by gray shading, with limits of alignment indicated by wavy lines. Note that alignment between proteins outside the identified domain is not shown.

Humans have three members of the FAM161 family (FAM161A/B/C, 569-660 residues), which overlap to a varying extent with the UPF0564/PF10595 domain ( Figure 1). FAM161A and FAM161B, are vertebrate-only paralogues, the human proteins being 31% identical (+16% homologous) across the region of 403 residues that matches 90% of UPF0564/PF10595 ( Bandah-Rozenfeld *et al.*, 2010). The remainder of this study will focus on FAM161A, with description of FAM161B largely omitted because it is so similar (data not shown). FAM161C (also called testis-specific protein 10-interacting protein, TSGA10IP) in only found in a few mammals. It contains ~40% of the C-terminus of UPF0564/PF10595, and is 29% identical (+24% homologous) to FAM161A across 227 residues in six blocks. This makes it an open question of whether FAM161C has a different function.

Databases created with profile-search tools report UPF0564/PF10595 homologues to be universal in animals, and also to be present in a minority of fungi, protists and plants, indicating that UPF0564/PF10595 has a conserved, fundamental function beyond photoreceptors. Profiles of FAM161A were created to prepare for profile-profile searches. A PSI-BLAST search identified known algal homologues on the first iteration. On the second iteration not only were known fungal homologues added, but also several large proteins annotated as titin ( Table 1). These positive alignments were based on large numbers of charged residues dispersed across the whole of FAM161A ( *Extended data:* Supplementary Figure 1A). This makes it very likely the hits represent non-specific false positives, which can occur with PSI-BLAST ( Schaffer *et al.*, 2001). In the third iteration, most new hits were titins and further iterations produced massive increases in hits, which were mostly long and repetitive proteins ( Table 1). This shows that a profile of FAM161A and its homologues cannot be built using PSI-BLAST with standard settings.

**Table 1.** Growth of numbers of sequences aligned to FAM161A in iterative searches.

search	iteration	1	2	3	4	5	6	7	8	9	10
1. PSI-BLAST (nr50)	hits	214	340	389	2357						
	other: Titin	0	x3 e-5	x29 e-24	x240 e-28						
2. PSI-BLAST (nr30)	hits	148	266	370	903	5285					
	other: Titin	0	x8 e-9	x66 e-20	x192 e-25	x403 e-29					
3. PSI-BLAST (filter low complexity) (nr100)	hits	1371	1483	1477	1480	1480	1484	=			
	other: Tpx2	0	x1 e-3	0	0	0	0				
4. HHblits (nr30)	hits	139	259	277	283	286	288	291	291	290	=
	other: Tpx2	0	x1 e-5	x1 e-6	x1 e-6	x1 e-6	x1 e-6	x1 e-6	x1 e-6	x1 e-6	

The numbers of hits with significant homologies (cut-off e-value ≤0.001) are shown for each iteration using four different strategies. 1/2: PSI-BLAST without a filter searching non-redundant databases of different sizes (nr50 and nr30 for #1 and #2 respectively); 3: PSI-BLAST with low complexity filter searching a comprehensive database (nr100); 4. HHblits searching a non-redundant database (nr30). Also showing the number of hits annotated as either titin for #1 and #2 or Tpx2 for #3 and #4 also showing e-value of hit. Red shading indicates multiple sequence alignment was too large for more iterations. Blue shading indicates convergence, with the next iteration identical.

Two different approaches were used to circumvent this problem. The first was to implement PSI-BLAST with a filter for regions of low complexity, which excluded 9 blocks in FAM161A ( *Extended data*: Supplementary Figure S1B). Multiple sequence alignments of this search barely grew after the second iteration and converged after the sixth iteration ( Table 1). The second approach was to use HHblits, which uses hidden Markov models (HMMs) to build the profile ( Remmert *et al.*, 2012). While traditional profile tools apply fixed rules on gap opening and deletions, HMMs develop alignment-specific rules. Using HHblits, the profile for FAM161A almost reached its final size at the third iteration, and converged at the ninth iteration.

### Profile-profile searches

**Figure 2.**
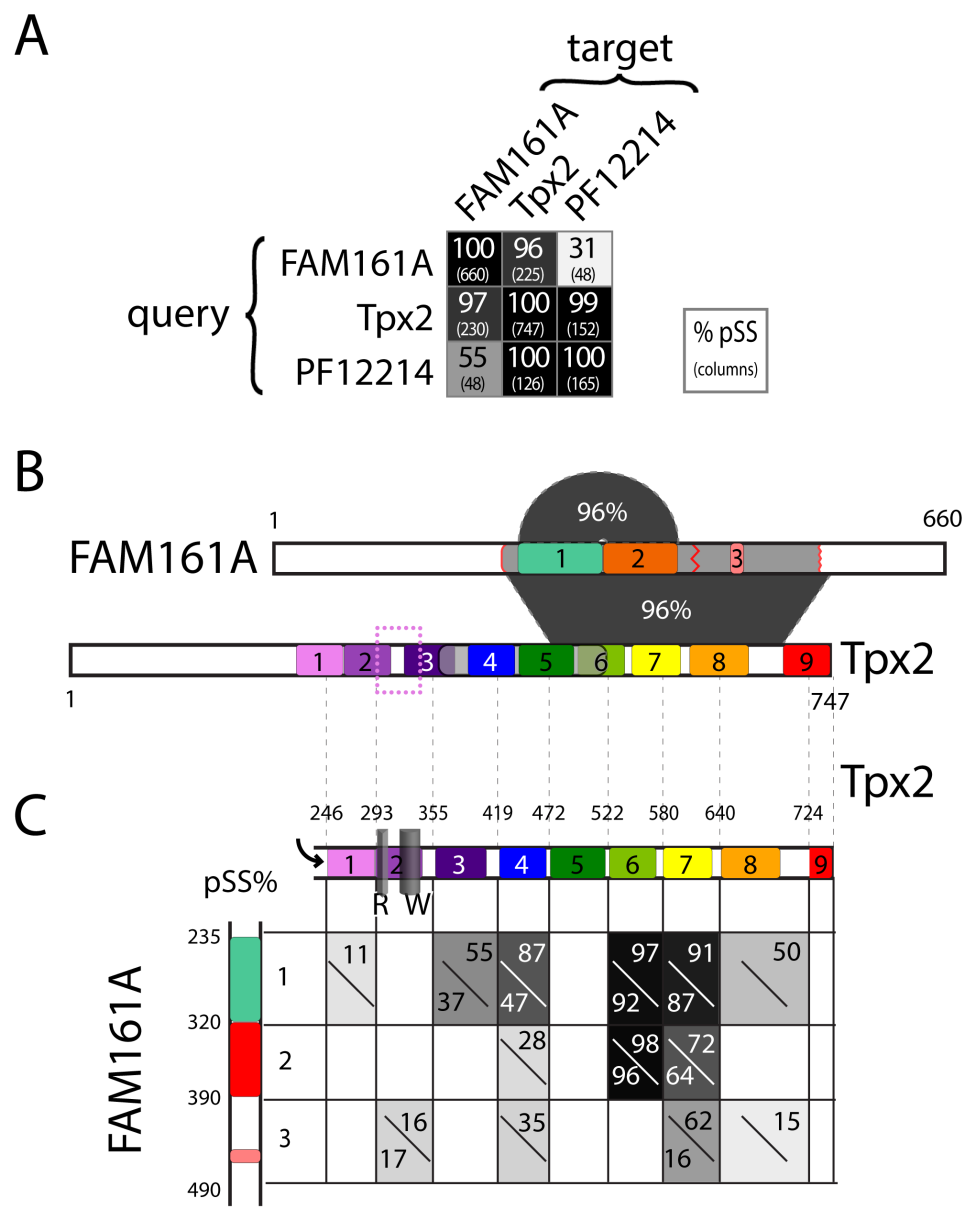
Fam161A and Tpx2 are homologous and contain repeats. ( **A**) Results of pair-wise HHpred searches between FAM161A, Tpx2 and the “Tpx2_importin” domain (PF12214, which covers residues 362-498 of Tpx2- see part B). The probability of shared structure (pSS, as %) and the number of columns (in brackets below) are reported for all pairwise searches. Results in PDB are identical to the human proteome, because Tpx2 is present in PDB as 6BJC_T with no additional DSSP secondary structural information. NB: self-searches (long diagonal) have pSS=100% and include all columns. ( **B**) Alignment of FAM161A with Tpx2. 320 residues of FAM161A (229–548) align with 228 residues of Tpx2 (474-701), which spans repeats 5 to 8. The probability of shared structure (pSS) =96%. Two adjacent repeats in FAM161A (79 and 88 residues, 239-317 and 318-395, both within the portion homologous to UPF0564) also align (pSS=96%, shaded semi-circle). Tpx2 repeats are coloured in a rainbow from violet to red. The region included in a cryo-EM structure with tubulin (residues 300-341) is shown by the dotted purple box. The region included in PF12214 is shown by the grey transparent box from repeat 3 to 6. ( **C**) pSS of pairwise comparisons of each repeat in FAM161A and Tpx2, where repeat boundaries are 25 residues down-stream of those in **B** (arrow). Shading of each cell represents the average pSS of two searches on a grey scale with pSS from 10-100% repesented by grey 10-100%, with FAM161A as query (bottom left); and with Tpx2 as query (top right). The structures labelled R and W in repeat 2 of Tpx2 are the ridge and wedge that bind tubulin (see Figure 3).

The FAM161A HHblits profile was submitted to the profile-profile search tool HHsearch, which is implemented online at the Tuebingen Toolkit as HHpred ( Soding, 2005; Soding *et al.*, 2005; Zimmermann *et al.*, 2018). HHpred was set to examine homologues in three databases: solved protein structures (PDB), PFAM, and the human proteome. There was a single significant hit: Tpx2, which stands for **t**argeting **p**rotein for ***X***enopus kinesin-like protein- **2** (Xklp2) ( Figure 2A). The hit was strong, with predicted shared structure (pSS) ≥95%, and 225 residues were matched (detail in Extended data: Supplementary Figure 2A). This is highly significant, as there were no false positives with hits of this strength in a benchmarking study ( Fidler *et al.*, 2016). A similar hit (pSS=98%) was obtained submitting the non-converged profile from the second iteration of PSI-BLAST (data not shown). The fully converged PSI-BLAST profile was too large to submit. Reverse searches, seeded with Tpx2 or its internal domain “Tpx2_importin” (PF12214), showed the same homologies to FAM161A/UPF0564 ( Figure 2A), which is important as profile-profile searches can sometimes produce asymmetric results ( Levine, 2019).

The aligned region of FAM161A was residues 229–548; the region of Tpx2 that matched it was 474–701 ( Figure 2B). Tpx2 has previously been shown to contain nine repeats of length 45–60 residues occupying its final two thirds (residues 222–747) ( Sanchez-Pulido *et al.*, 2016). The alignment covered from the C-terminal half of repeat 5 to the start of repeat 9. FAM161A also produced out-of-register hits with itself, indicating the presence of two repeats of ~70 residues (237–312 and 320–385, Figure 2B). In addition, a partial third repeat was found ~60 residues downstream (447-463) ( Figure 2B, and see below). Using a non-default setting designed to increase accuracy of alignments, the Maximum Accuracy algorithm (MAC), repeats 1 and 2 only (FAM161A 236-361) showed homology to four regions of Tpx2, the strongest focussed on repeat 6, and others shifted either one or two whole repeats further on, or two repeats earlier ( *Extended data*: Supplementary Figure S2B).

Since repeats can occasionally align out of register, reducing the specificity for individual repeats, all repeats from FAM161A and Tpx2 were compared against each other. This showed that repeat 1 of FAM161A is most like Tpx2 (particularly its repeats 4/6/7), followed by repeat 2 of FAM161A and then repeat 3 ( Figure 2C). In addition, the presence of non-conserved unstructured regions just prior to each repeat in FAM161A and in the centres of each repeat in Tpx2 suggest that the boundaries of Tpx2 repeats should be drawn in a different register that matches FAM161A, approximately 25 residues further toward the C-terminus than previously ( Sanchez-Pulido *et al.*, 2016). This shortens repeat 9, which shows no homologies to FAM161A, as might be expected given its well-defined function to bind to kinesin ( Eckerdt *et al.*, 2008). Homologies detected between individual repeats might be revealing. Among the Tpx2 repeats, repeat 2 is an outlier. While all the others are homologous to 3 others, it shows homologies only to itself (data not shown). However, it does show weak homology to a single region of FAM161A in repeat 3 ( Figure 2C).

Finally, a re-examination of the profile-sequence searches supported the homology between FAM161A and Tpx2 homology: PSI-BLAST and HHblits both produced occasional significant hits to Tpx2 ( Table 1, rows 3 and 4); also a large proportion of the weak hits marginally outside the inclusion cut-off are Tpx2 (data not shown).

Overall, these results strongly predict that FAM161A is a homologue of Tpx2.

*FAM161A has three repeats of the extended loop in Tpx2 that nucleates microtubules*

**Figure 3.**
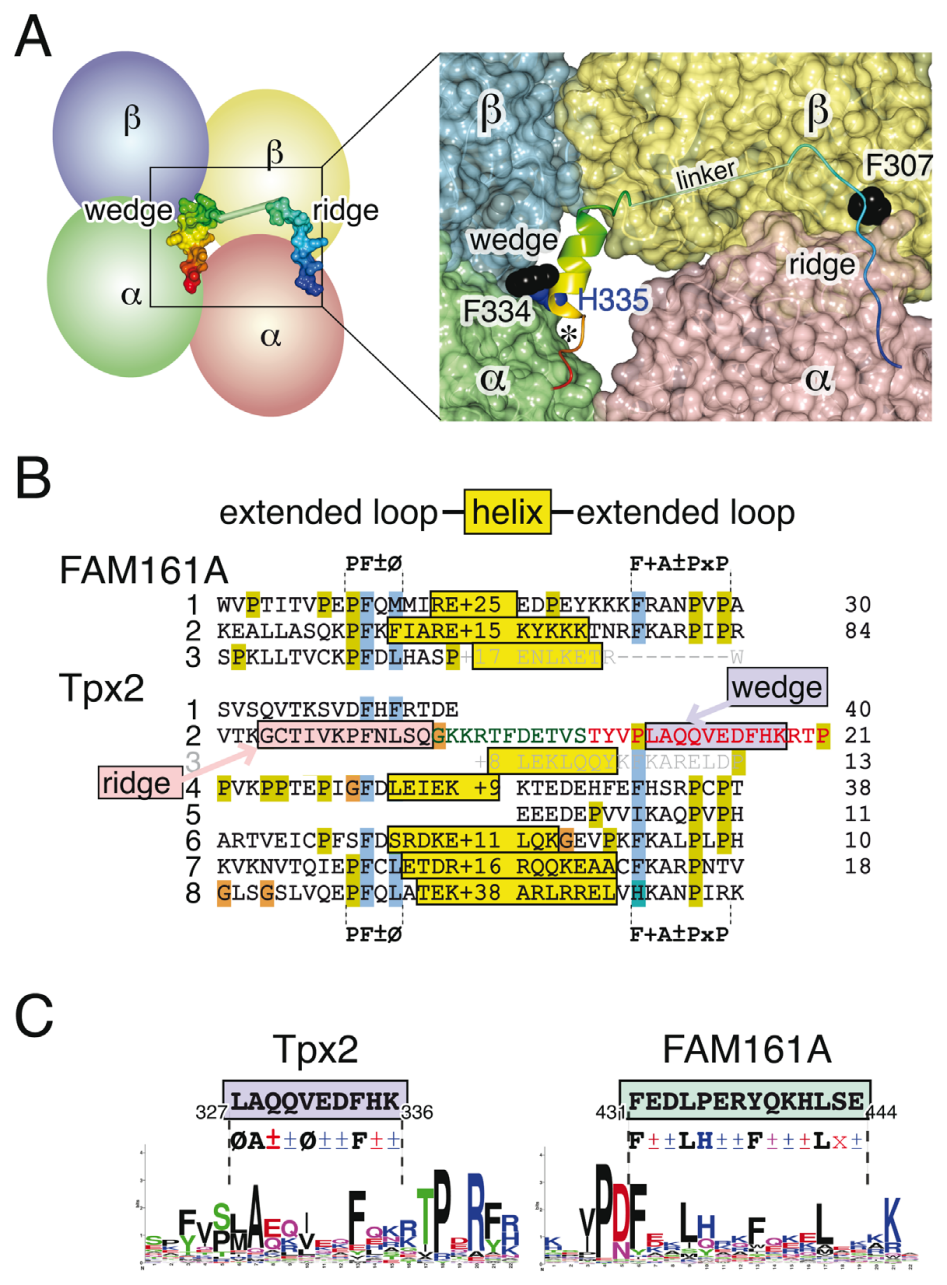
FAM161A has elements like the ridge and wedge of Tpx2. ( **A**) Left: scheme of binding of one copy of Tpx2 residues 300-341 (rainbow blue→red) binding to four tubulin monomers. Right: close up of the surfaces of the tubulins and Tpx2, shown as a ribbon, with the ridge (300-311) and wedge (323-341), but residues 312-322 modelled as a straight rod (length 2.4 nm) as they not seen in the cryo-EM structure. Conserved side-chains in Tpx2 (F307 F334: black, H335: blue) bind into pockets formed at tubulin interfaces. Image derived from PDB 6BJC chains A-D and P ( Zhang *et al.*, 2017). The asterisk indicates space at the C-terminus of the wedge (see Discussion). ( **B**) FAM161A and Tpx2 contain repeated ridge-like motifs. Left: Ungapped alignment of extended loops from repeats 1–3 of FAM161A and repeats 1–8 of Tpx2, indicating PF±Ø motifs. Pink box indicates the ridge sequence in repeat 2. Sequence directly after repeat 2 is also included, showing the linker (green letters) and wedge (red letters, helix in blue box). Centre: yellow boxes indicate charged predicted helices (average length 25 aa, numbers of missing residues shown). Right: Extended loops following the helices, containing FKA±PxP motifs. The analogous sequences in repeat 3 of both proteins do not form motifs, but are included for comparison (grey letters). Shading is according to the Clustal scheme. Numbers on the right indicate omitted residues between repeats. ( **C**) Comparison of the wedge helix in Tpx2 with the amphipathic helix in FAM161A. Both the wedge of Tpx2 (blue box, left) and the predicted amphipathic helix of FAM161A (green box, right) are accompanied by logos made from 40 diverse sequences, and by a consensus in which bold lettering indicates a strong conservation, Ø is hydrophobic (black), ± is charged or polar, and other colouring is: D/E-red, KRH-blue, Q-purple, A-black.

Tpx2 is a microtubule-associated protein (MAP) ( Wittmann *et al.*, 1998), as is FAM161A ( Zach *et al.*, 2012). While the interaction with microtubules by FAM161A is not understood in detail, the tubulin–Tpx2 interaction has been studied by cryo-EM in ultrastructural detail at 3.3 Angstrom resolution ( Zhang *et al.*, 2017). Two short structural elements in Tpx2 in a region of 42 residues starting in repeat 2 (300-341, purple box in Figure 2B) contact four tubulins, showing how Tpx2 can stimulate microtubule nucleation ( Figure 3A, left) ( Zhang *et al.*, 2017). This region coincides with the redrawn boundaries of repeat 2 ( Figure 2C). Further analysis is required to determine if the homology uncovered by HHpred between this repeat and repeat 3 of FAM161A is consistent with FAM161A having elements that crosslink tubulin.

The first microtubule nucleating element in repeat 2 of Tpx2 is the extended loop __300__GCTIVKPFNLSQ __311__, which forms a “ridge” that runs parallel to protofilaments to crosslink α- and β-Tubulin. The conserved residue F307 binds in a hydrophobic pocket formed by both tubulins ( Figure 3A, right) ( Zhang *et al.*, 2017). Profile-profile searches showed that five other Tpx2 repeats (1/4/6/7/8) contain ridge-like sequences, which are characterised by Pro-Phe-charged/polar-hydrophobic (PF±Ø) motifs ( Figure 3B, left). The motif is missing from repeats 3 and 5, although other aspects of these repeats are preserved ( Figure 3B, right).

FAM161A repeats 1 and 2 both contain ridge-like sequences with PF±Ø. In addition, a third ridge-like sequence was found in FAM161A ~80 residues beyond C-terminus of repeat 2, and this was therefore designated as a partial third repeat. This region of FAM161A is homologous to FAM161C ( *Extended data*: Supplementary Figure S2B), and although FAM161C lacks the key phenylalanine ( __336__PQKL __339__), a variant PF±Ø motif may lie upstream ( __318__PWDL __321__).

Ridge-like sequences in Tpx2 repeats 4–8 run directly into helices ( Alfaro-Aco *et al.*, 2017). The helices are on average 25 aa, and they are highly enriched for charged residues. The charged helices are followed by unstructured loops that contain characteristic FKA±PxP motifs ( Figure 3B, centre and right), which have been noted previously ( Alfaro-Aco *et al.*, 2017). Significantly, FAM161A repeats 1 and 2 both have the same loop-charged helix-loop form ( Figure 3B). Therefore, overall FAM161A contains three copies of sequences that align without gaps to the ridge in Tpx2, which is itself repeated six times. Two of the FAM161A repeats and four of the Tpx2 repeats have the same arrangement, namely loop (ridge-like, PF±Ø)–charged helix–loop (FKA±PxP).

*FAM161A contains an amphipathic helix that resembles the “wedge” of Tpx2*

A second element in Tpx2 that nucleates microtubules is a short helix (10 residues) that has been described as a “wedge”. This fits into a deep pocket that forms between four tubulin monomers between side-by-side protofilaments ( *Extended data*: Supplementary Figure S3) ( Zhang *et al.*, 2017). The helix is amphipathic, having hydrophobic residues in positions 1/5/8 ( *i.e.* all on the same face), with charges or large polar residues at most other positions ( Figure 3C). Two conserved residues on the helix ( __334__FH __335__) contribute to microtubule nucleation by binding into a hydrophobic pocket formed by two tubulin monomers ( Figure 3A). The wedge is separated from the ridge by an 11 aa linker, which is long enough to reach across one β Tubulin molecule between the two different interfaces ( Figure 3A). Unlike the ridge which is repeated six times, Tpx2 only contains one short amphipathic helix, as indicated by a higher hydrophobic moment compared to the charged helices in the other repeats ( *Extended data*: Supplementary Figure S4) ( Gautier *et al.*, 2008).

While one of the five helices found in repeats with ridge-like sequences is amphipathic, neither of the two helices in FAM161A repeats are amphipathic ( *Extended data*: Supplementary Figure S4). However, another location in FAM161A, immediately before the third ridge-like sequence, does contain a predicted amphipathic helix ( *Extended data*: Supplementary Figure S2). This is the only region of FAM161A that shows specific homology to repeat 2 of Tpx2 ( Figure 2C). While this helix is 14 residues, making it one turn longer than the helix in Tpx2, it is otherwise similar, with regularly spaced hydrophobic residues, including a phenylalanine at the 8th position, as found in Tpx2 ( Figure 3C). This region is widely conserved in FAM161A homologues across evolution ( *Extended data*: Supplementary Figure S5), though there are some exceptions: in mouse the FAM161A helix is shorter, and in trypanosomes the key aromatic side chain is missing. FAM161B and FAM161C contain variations of this amphipathic helix ( __375__YEGLYKAFQRRAAK __388 __and __321__LEKLHRQLQRDL __332__ respectively). In FAM161C this overlaps the PF±Ø-like motif identified above, so binding of the helix and the ridge would be mutually exclusive.

Thus, FAM161A has a sequence that has many properties of the wedge in Tpx2, except for making one additional helical turn.

## Discussion

Here, FAM161A is shown to contain repeats similar to those in Tpx2. Since the way Tpx2 interacts with microtubules is now understood in great detail ( Zhang *et al.*, 2017), it has been possible to show that the sequence of FAM161A shares most of the requirements to bind microtubules too, in particular having three ridge-like extended loops, as well as a more variant wedge-like helix. 

The residues of both the ridge and wedge of Tpx2 fit precisely into the surface of tubulin. This suggests that ridges and wedges may not tolerate gaps or insertions, and is consistent with the strongly conserved pattern found in ungapped alignments of FAM161A ( *Extended data*: Supplementary Figure S5A). The homology might be missed if gaps are tolerated during alignment ( Sanchez-Pulido *et al.*, 2016). The application of HMMs, which adjust these parameters for each search, might underlie the considerable gain in sensitivity obtained here.

The main outstanding question is whether the FAM161A helix is functionally like the wedge in Tpx2. Given the requirement to bind onto the surface of tubulin, which is highly conserved, the additional full turn in the only predicted amphipathic helix in FAM161A might be expected to prevent it fitting into the deep pocket in tubulin, despite it sharing many biochemical properties with the wedge of Tpx2 ( Figure 3C). However, the wedge in some Tpx2 homologues is also predicted to be extended by one turn, as they lack the helix-breaking proline ( *Extended data*: Supplementary Figure S6). On this point, the tubulin structure leaves unfilled space room beyond the C-terminus of the Tpx2 wedge (asterisk in Figure 3A and *Extended data*: Supplementary Figure S3B). Another piece of evidence is that repeat 2 in Tpx2 shows homology only to repeat 3 of FAM161A. Although this alignment is based solely on the ridge ( Figure 2C and data not shown), it suggests that the nearby amphipathic helices may share a function. One way forward to examine this question would be to study protein docking *in silico*. As for the finding that the proposed ridges and wedge in FAM161A are not arranged in the same relationship as found in Tpx2, such differences exist among Tpx2 homologues. For example, in algae the wedge precedes all ridge repeats (data not shown). If it were determined that the FAM161A helix cannot act as a wedge, it is worth noting that Tpx2 homologues in some species do not have any wedge: for example, none of the three short Tpx2 homologues in flies have one (data not shown) ( Sanchez-Pulido *et al.*, 2016).

**Figure 4.**
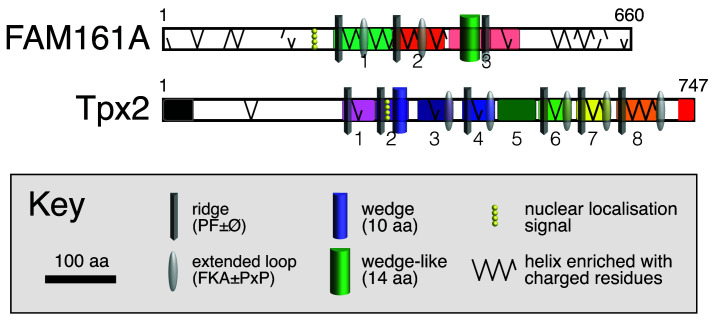
Structural and functional elements in FAM161A and Tpx2. Sequence and structural motifs as shown in key: prism = loop similar to ridge with PF±Ø; ovoid = loop with FKA±PxP; half cylinder = amphipathic helix: wedge–blue, FAM161A–green; yellow spheres: NLS; zig-zag = all charged helix. Repeat boundaries shown as in Figure 2C, with two known binding sites in Tpx2: aurora kinase A at N-terminus (black) and kinesin at C-terminus (red).

One of the first molecular studies of FAM161A described it as a MAP ( Zach *et al.*, 2012). At endogenous levels, it localises to the basal body at the base of the primary cilium and in the inner segment which contains a mixture of organelles ( Di Gioia *et al.*, 2012). When over-expressed it localises to microtubules. Its 140 interactors detected by yeast two-hybrid are enriched for residents of the cilium ( Di Gioia *et al.*, 2012) and also residents of the Golgi, centrosome and microtubules ( Di Gioia *et al.*, 2015). Two centrosomal interactors are well described: **t**ransforming **a**cidic **c**oiled- **c**oil-3 (TACC3) ( Gomez-Baldo *et al.*, 2010), and **p**roteome **o**f the **c**entriole-1B (POC1B, also called WDR51B) ( Di Gioia *et al.*, 2015). The centriole-specific function of FAM161A is now understood in ultrastructural detail, and involves the interaction with POC1B, as well as POC5 and centrin form a complex that multimerises into a cylindrical scaffold adhering to the inside of centrioles ( Le Guennec *et al.*, 2020). This scaffold is different from the centriole’s internal SAS-6 cartwheel that projects radially from the central axis ( Gonczy, 2012). FAM161A plays a central role, binding not only the three other components, but also tubulin. The molecular basis for that binding has previously been unknown, but the findings here provide models for this interaction. Given that another cylindrical layer containing WDR90 is proposed to lie between the FAM161A layer and microtubules ( Steib *et al.*, 2020), FAM161A may only be able bind microtubules through gaps in the WDR90 layer. This might explain how evolution has produced small microtubule-binding domains of short loops and helices ( Figure 4).

The precise function of FAM161A can be interpreted by comparing its overall form with Tpx2 ( Figure 4). Tpx2 has three separate microtubule binding sites: (1) the N-terminal 240 residues, which includes the extreme N-terminal Aurora A kinase binding site ( Brunet *et al.*, 2004); (2) residues 236-352 (repeats 1&2, including ridge and wedge) ( Trieselmann *et al.*, 2003); (3) residues 547-579 (repeat 6) and an undefined amount of flanking sequence ( *i.e.* ridge repeats, possibly more than one) ( Trieselmann *et al.*, 2003). A truncation of six repeats (2–7, residues 274-659) can nucleate microtubules, albeit sub-optimally ( Roostalu *et al.*, 2015). Maybe the three repeats in FAM161A can act in the same way. Tpx2 is regulated by RanGTP ( Gruss *et al.*, 2001), but this is not likely to occur in FAM161A. The NLS in Tpx2 overlaps the ridge, so that binding to importin and tubulin are mutually exclusive ( Giesecke & Stewart, 2010). In contrast, the predicted NLS in FAM161A (224-230) is not near the tubulin binding sites ( Figure 4). The second extended loop found after the charged helix in most repeats of both Tpx2 and FAM161A has yet to be studied, but its conserved motif suggests a specific interaction ( Alfaro-Aco *et al.*, 2017), which might be with tubulin, other copies of Tpx2 or another MAP in stoichiometric complexes.

In summary, the homologies discovered here ( Figure 2) strongly predict that FAM161A is a previously unknown homologue of Tpx2. This could underlie its binding to tubulins in centrioles, and also allow the pool of FAM161A in other parts of the cell, possibly the Golgi apparatus ( Di Gioia *et al.*, 2012), to be a microtubule nucleation factor. The homologies produce testable models to study the function of FAM161A by directed mutagenesis of residues that align with Tpx2.

## Data availability

### Underlying data

UniProtKB - Q3B820 (F161A_HUMAN), Accession number Q3B820: https://www.uniprot.org/uniprot/Q3B820

UniProtKB - Q9ULW0 (TPX2_HUMAN), Accession number Q9ULW0: https://www.uniprot.org/uniprot/Q9ULW0

### Extended data

Harvard Dataverse: Extended data, https://doi.org/10.7910/DVN/EVAGZU ( Levine, 2020).

This project contains the file ‘Supplementary figures.pdf’, which contains the following extended data:

- Supplementary Figure S1: A. Hit to titin in the second iteration of PSI-BLAST into the nr50 database; B. Low complexity regions in FAM161A.
- Supplementary Figure S2: A. HHpred result of symmetrical pairwise alignment of Fam161A and Tpx2; B. HHpred search with alignment realigned with Maximum Accuracy algorithm
- Supplementary Figure S3: The wedge helix of Tpx2 is buried deeply in the pocket formed by four tubulin monomers
- Supplementary Figure S4: Properties of helices following ridge sequences in Tpx2 and FAM161A
- Supplementary Figure S5: Sequences in amphipathic helices in the FAM161 family
- Supplementary Figure S6: Variation of Tpx2 ridge and wedge sequences across species.

Data are available under the terms of the Creative Commons Zero "No rights reserved" data waiver (CC0 1.0 Public domain dedication).
