# Supplementary material for: Structural bioinformatics predicts that the Retinitis Pigmentosa-28 protein of unknown function FAM161A is a homologue of the microtubule nucleation factor Tpx2
Source: F1000Res. 2020 Aug 27;9:1052. [Version 1] doi: 10.12688/f1000research.25870.1 (PMC7551519; doi:10.12688/f1000research.25870.1)
Supplement: Peer review history

# Peer review: Reviewer response for version 1

**DOI:** 10.5256/f1000research.28550.r70565

**Published:** 2020-10-12

**Authors:** Paul Guichard

**Affiliations:** 1 Department of Cell Biology, Sciences III, University of Geneva, Geneva, Switzerland

Timothy Levine's research paper is a bioinformatic analysis of the FAM161a protein. This study identified regions within FAM161A homologous to the Tpx2 protein. FAM161a is a microtubule-associated protein that is localized at the centrioles/basal bodies, Golgi apparatus, and at the connecting cilium of the photoreceptors. Truncations of this protein cause retinitis pigmentosa, a disease leading to photoreceptor degeneration and subsequent blindness.

 Since this protein is poorly studied, this bioinformatic analysis is important because it defines potential functional domains for this protein. This analysis can be used as a basis for future in-depth biochemistry and structural biology studies.

 Computer databases indicate that a domain called UPR0564/PF10595 exists in this protein, as well as in its homologs FAM161A and FAM161C. However, no function has been attributed to this domain. From that point on, T. Levine used PSI-Blast and hidden Markov models (HMMs) approaches to find homologs. Importantly, HMMs identified TPX2, a well-characterized microtubule-binding protein that contains nine repeats of length 45–60 residues, as a putative homolog. In addition, T. Levine identified two to three repeats in the sequence of FAM161A of about 60-70 residues that showed homology to four regions of TPX2. Further analysis, using the known structural interaction of TPX2 with microtubules, leads to propose that FAM161A homologous sequence contains three ridge-like extended loops, as well as a more variant wedge-like helix, structural domains that interact with microtubules and therefore are potentially crucial for FAM161A function.

 This bioinformatics analysis is very interesting and reveals a surprising homology with TPX2. Thanks to this work, T. Levine defines potential functional domains of this protein. This work is a crucial starting point to study the structure and function of FAM161A in order to better understand the mechanisms that leads to retinitis pigmentosa.

Is the work clearly and accurately presented and does it cite the current literature?

Yes

If applicable, is the statistical analysis and its interpretation appropriate?

Yes

Are all the source data underlying the results available to ensure full reproducibility?

Yes

Is the study design appropriate and is the work technically sound?

Yes

Are the conclusions drawn adequately supported by the results?

Yes

Are sufficient details of methods and analysis provided to allow replication by others?

Yes

Reviewer Expertise:

Cell Biology

I confirm that I have read this submission and believe that I have an appropriate level of expertise to confirm that it is of an acceptable scientific standard.

# Peer review: Reviewer response for version 1

**DOI:** 10.5256/f1000research.28550.r70563

**Published:** 2020-10-01

**Authors:** Vikram Alva

**Affiliations:** 1 Department of Protein Evolution, Max Planck Institute for Developmental Biology, Tübingen, Germany

This article by Timothy Levine substantiates a homologous relationship between the microtubule-associated ciliary protein FAM161A and the microtubule-associated spindle assembly factor Tpx2. While the interaction between Tpx2 and microtubules has been characterized structurally using cryo-EM, the interaction between FAM161A and microtubules remains poorly understood. Based on the conservation of microtubule-binding elements of Tpx2 in FAM161A, the author proposes that both these proteins bind microtubules in a similar fashion.

 The homologous connection between FAM161A and Tpx2, although distant, is well substantiated. The manuscript is very well written, and I have only some minor comments.

1. Page 3, Methods section: " *PSI-BLAST with low complexity filter was carried out at NCBI using the Refseq database (nr100, but 40% smaller than the database called “nr”)"*.
  Refseq is not the same as nr100! 
  https://www.ncbi.nlm.nih.gov/books/NBK50679/#RefSeqFAQ.what_is_a_reference_sequence_r
2. Page 3, Methods section: " *HHblits was used iteratively in the same way, except all queries used the nr30 database*".
  I believe the HHblits searches reported in this study were carried out against the Unclust30 database and not the nr30 database; this also needs to be corrected in Table 1.
3. The author refers to the probability values yielded by HHpred as the probability of shared structure (pSS). This is, however, incorrect. While the sequence similarity of proteins reflects homology, structural similarity alone may often be analogous because only a limited number of folded conformations are available to the polypeptide chain. Consequently, proteins unrelated in sequence may exhibit substantially similar structures. However, in such cases, HHpred typically does not report high probability values. The probability value reported by HHpred is an estimate for a match to be a true positive. It allows inferring if an obtained match is homologous to the query or is just a chance hit.
4. Page 4, paragraph 1: " *FAM161A and FAM161B, are vertebrate-only paralogues, the human proteins being 31% identical (+16% homologous) across the region of 403 residues that matches 90% of UPF0564/PF10595*".
  It is unclear what the author means by 16% homologous. Sequence identity can be quantified, but homology is a qualitative description of the relationship between proteins and cannot be quantified.
5. FAM161A and Tpx2 appear to contain multiple coiled-coil segments. It would be useful to the readers if the author could comment on this in the text. 
6. Are the microtubule-binding elements observed in Tpx2 and FAM161A also present in FAM161B and FAM161C? The author could consider including a few sentences on this in the text.

Is the work clearly and accurately presented and does it cite the current literature?

Yes

If applicable, is the statistical analysis and its interpretation appropriate?

Yes

Are all the source data underlying the results available to ensure full reproducibility?

Yes

Is the study design appropriate and is the work technically sound?

Yes

Are the conclusions drawn adequately supported by the results?

Yes

Are sufficient details of methods and analysis provided to allow replication by others?

Yes

Reviewer Expertise:

Protein bioinformatics and evolution

I confirm that I have read this submission and believe that I have an appropriate level of expertise to confirm that it is of an acceptable scientific standard.
